# The Heterogeneous Allelic Repertoire of Human Toll-Like Receptor (*TLR*) Genes

**DOI:** 10.1371/journal.pone.0007803

**Published:** 2009-11-17

**Authors:** Philippe Georgel, Cécile Macquin, Seiamak Bahram

**Affiliations:** 1 Laboratoire d'Immunogénétique Moléculaire Humaine, Centre de Recherche d'Immunologie et d'Hématologie, Faculté de Médecine, Université de Strasbourg, Strasbourg, France; 2 Faculté de Pharmacie, Université de Strasbourg, Illkirch-Graffenstaden, France; 3 Laboratoire Central d'Immunologie, Plateau Technique de Biologie, Nouvel Hôpital Civil, Hôpitaux Universitaires de Strasbourg, Strasbourg, France; University of California Merced, United States of America

## Abstract

Toll-Like Receptors (TLR) are critical elements of the innate arm of the vertebrate immune system. They constitute a multigenic family of receptors which collectively bind a diverse array of – exogeneous as well as endogeneous – ligands. An exponential burst of knowledge has defined their biological role in fight against infections and generation/modulation of auto-immune disorders. Hence, they could at least be conceptually recognized – despite being structurally unrelated – as innate counterparts to Major Histocompatibility Complex (MHC) molecules – equally recognizing antigenic ligands (albeit structurally more homogeneous i.e., peptides), again derived from self and/or non-self sources – preeminent this time in adaptive immunity. Our great disparities in face of infections and/or susceptibility to auto-immune diseases have provoked an intense search for genetic explanations, in part satisfied by the extraordinary MHC allelic repertoire. An equally in-depth and systematic analysis of *TLR* diversity is lacking despite numerous independent reports of a growing number of SNPs within these loci. The work described here aims at providing a preliminary picture of the allelic repertoire – and not purely SNPs – of all 10 human TLR coding sequences (with exception of TLR3) within a single cohort of up to 100 individuals. It appears from our work that TLR are unequally polymorphic: TLR2 (DNA alleles: 7/protein alleles: 3), 4 (4/3), 7 (6/3), 8 (9/2) and 9 (8/3) being comparatively least diverse whereas TLR1 (11/10), 5 (14/12), 6 (10/8) and 10 (15/10) show a substantial number of alleles. In addition to allelic assignment of a large number of SNPs, 10 new polymorphic positions were hereby identified. Hence this work depicts a first overview of the diversity of almost all human TLR genes, a prelude for large-scale population genetics as well as genetic association studies.

## Introduction

In collaboration with other pattern recognition Receptors, Toll-like Receptors (TLRs) govern the innate arm of the immune system. The first member of this family, TOLL, was originally described through its function in the *Drosophila* embryonic development and was later found to be involved in the response against Gram positive bacteria and fungi within this organism [1]. This discovery prompted the *in silico* identification of mammalian orthologues. A positional cloning approach of the Lps^d^ locus led to the identification of the mouse *TLR4* and to its description as the LPS sensor which is essential for an efficient response against Gram-negative bacteria [2]. These findings emphasized the evolutionary conservation of the TLRs and suggested that these molecules play a fundamental role in the early detection of pathogens and the appropriate subsequent innate immune response (reviewed in [3], [4]).

To date, 10 *TLRs* have been described in humans (12 in mice) dispersed all over the genome. The molecular identification of their respective ligands, which has been unraveled in most cases, revealed that TLRs allow detection of virtually any potential pathogen and a number of self-entities. As such, they constitute an “innate early repertoire”, whose importance is confirmed by the increasing amount of micro-organisms that are sensed by the TLR system, as evidenced by the susceptibility phenotypes observed when TLR-deficient animals are challenged by various pathogens. The molecular diversity of the motifs recognized by the different TLRs is an intriguing feature (discussed in [5]). Despite structural homogeneity (defined by the presence of extracellular leucine-rich repeats, a transmembrane domain and a Toll-Interleukine 1 Receptor - TIR - region), TLRs are able to sense the presence of unrelated molecules such as triacetylated lipopeptides (TLR1/2), lipotechoic acid (TLR2/6), double-stranded RNA (TLR3), lipoploysaccharide (TLR4), flagellin (TLR5), single-stranded RNA (TLR7) or unmethylated double-stranded DNA (TLR9) to cite only a few ligands (reviewed in [6]). Heterodimerization between TLRs or association with co-receptors such as CD14 or CD36 clearly contributes to increasing the diversity of molecules that can be recognized by TLRs [7], [8]. However, this wide variety of ligands makes the TLRs a critical interface between invading pathogens and the host. In addition, the recent discovery that TLR signaling has a profound impact on the activation and shaping of the adaptive immune response has highlighted the importance of this field and explains its recent explosive development.

In humans, challenging questions regarding the role of TLRs in the defense against infectious diseases are complicated by environmental factors and multiple genetic differences between people. However, several studies have evidenced the association of specific polymorphisms in genes encoding TLRs and microbial infections. One of the well-described examples is the presence of D299G and T399I substitutions in TLR4 which causes decreased airway response to inhaled lipoloysaccharide in humans [9]. Since then, a large collection of reports has studied the association of these *TLR4* SNPs with infectious diseases (reviewed in [10] with conflicting conclusions [11]. Additional analysis of patients suffering from meningococcal infections established that rare mutations affecting TLR4 structure contribute to disease susceptibility [12]. Association of human diseases with polymorphisms in other TLRs have been investigated, including TLR2 [13], TLR3 [14] and TLR 5 [15]. TLR9, which classically senses DNA bearing unmethylated CpG motifs of bacterial or viral origins, also activates signaling when stimulated with mammalian DNA engaged within immune complexes [16]. This has led to the hypothesis that TLR9 might be implicated as risk factors in the development of autoimmune diseases such as systemic lupus erythematosus (SLE). Altogether, these studies have provided substantial evidence for an association between the presence of some SNPs in TLRs and the prevalence of infectious diseases. However, strong genetic association has never been achieved, mostly because the correlations were measured in small populations. To confirm the impact of TLRs in human health, more epidemiological studies and additional genomic analysis need to be performed. However prior to embarking in such vast investigations a clear picture of the allelic (and importantly not only SNP) repertoire of all TLR is needed. This is reminiscent somewhat to the situation with histocompatibility genes, which could conceptually (and only conceptually) be considered adaptive equivalents to the TLR or vice versa. The HLA genes are indeed highly polymorphic [17]. This polymorphism both serves as a primary tool for a population-wide protection against infectious agents and is involved in graft rejection and the still partly mysterious susceptibility to a large number of diseases. This study aims to contribute to bringing the TLRs “up-to-speed” with HLA genes.

## Results

### *TLR1*

**Figure 1:**
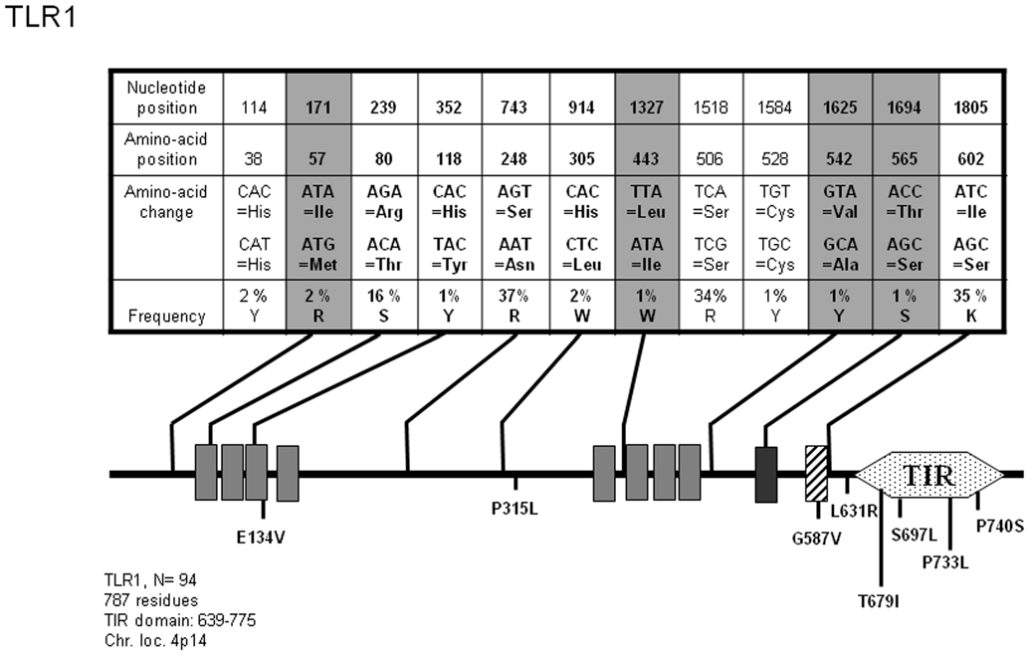
Schematic representation of TLR1 and position and frequency of Single Nucleotide Polymorphisms (SNPs). Non Synonymous (NS) SNPs are indicated in bold letters and those in grey boxes represent new NS-SNPs identified in this study. Leucine-Rich Repeats (LRR) are represented by grey boxes (dark grey for the C-terminus LRR). The trans-membrane (TM) domain is shown as a hatched box and the Toll-Interleukin 1 Receptor (TIR) domain as a dotted hexagon. The position of NS-SNPs recorded in databases but not identified in this study is indicated.

TLR1, when associated to TLR2 (see below) in a heterodimeric complex, is able to sense triacylated lipoproteins purified from mycobacteria or synthetic peptides such as N-palmitoyl-S-dipalmitoylglyceryl (Pam3) Cys-Ser-(Lys)4 (CSK4) [18]. To date, few pathologies have been genetically associated to TLR2 variants; an increased risk to develop prostate cancer has been traced to TLR1 (and TLR6 and 10 which are located on the same chromosomal locus, [19] and more recently, resistance to leprosy was linked to the frequent I602S TLR1 allele [20]. A search for *TLR1* SNPs in several databases (http://snpper.chip.org/bio/ and http://www.ncbi.nlm.nih.gov/SNP/) identified 13 mutations at positions leading to a non-synonymous amino-acid change. As illustrated in Fig. 1, our sequencing effort performed on 94 individuals allowed us to detect 5/13 of these known modifications and also, to characterize four additional mutations leading to a different amino-acid sequence of the TLR1 protein. According to the recently published crystallographic model of TLR1/2 bound to the PAM3CSK4 [21], L443I, V542A and T565S are located at the interface important for dimer formation. Therefore, such alterations which have the potential to impair not only TLR1/2, but also TLR1/10 association, could have a wider than suspected impact on immune defense. On the other hand, I57M mutation, localized at the N terminus of the TLR1 protein, could potentially affect the folding of the extracellular domain and thus, modify ligand recognition. These 4 SNPs were detected in 1 to 2% of the samples (N = 93 genomic DNAs tested for *TLR1*) and each of them define a new allele (see Table S1) which was observed at a low frequency (0.56% of the population among the 11 *TLR1* alleles which have been defined in this study). This allelotyping analysis also shows that the frequent I602S allele, which has been mostly studied so far, is usually associated with the S248N mutation and in 7.8% of the population, is also linked to the R80T SNP.

### *TLR2*

**Figure 2:**
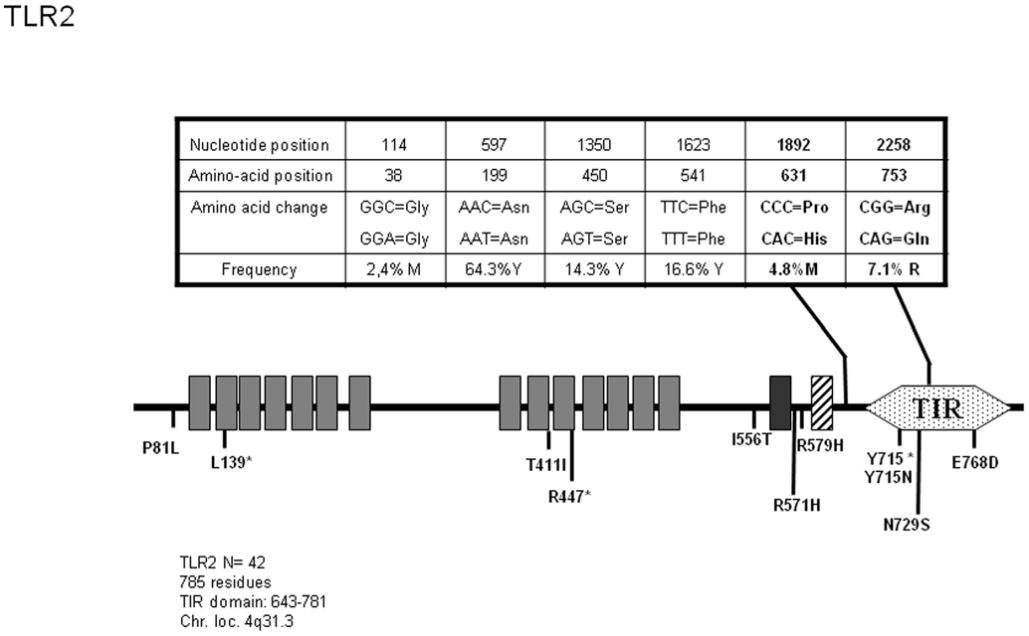
Schematic representation of TLR2 and position and frequency of Single Nucleotide Polymorphisms (SNPs). Legend is similar to Fig. 1.

Despite its reported association with TLR1, TLR2 variants are associated to a much wider panel of infectious diseases. These can be of viral [22], [23] or bacterial [24], [25] origin. Currently, 24 polymorphisms within the human *TLR2* gene are described, among which 13 are non-synonymous mutations leading to amino acid exchanges. We were able here to identify 2 already known SNPs affecting the TLR2 protein sequence (P681H and R753Q) in addition to 4 synonymous SNPs (Fig. 2). No novel SNP could be identified in the course of our sequencing of 42 samples. These 6 nucleotides changes served to define 7 alleles, but only 3 forms of the TLR2 protein (Table S2), those expressing P631H and R753Q representing respectively 2.33 and 3.49% of our population.

### *TLR4*

**Figure 3:**
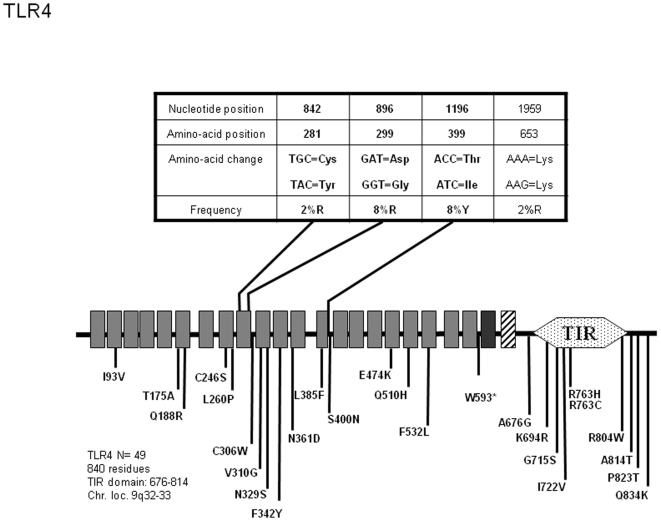
Schematic representation of TLR4 and position and frequency of Single Nucleotide Polymorphisms (SNPs). Legend is similar to Fig. 1.

Among TLRs, TLR4 appears to have been the subject of intense attention. 28 non synonymous (and 7 synonymous) positions have been described so far, among which two co-segregating mutations inducing D299G and T399I amino acid change have been studied with remarkable scrutiny (Fig. 3). A large body of genetic analysis suggests potential association between the presence of these frequent SNPs and several pathologic conditions of diverse etiology. Because of its role as a component (in addition to MD2 and CD14) of the LPS receptor, *TLR4* polymorphisms were mostly involved in susceptibility to Gram-negative bacteria infections. However, increased fungal or viral infections and non-infectious conditions such as atherosclerosis were also tentatively linked to the presence of the most frequent D299G and T399I SNPs [10], [26]. In several cases, however, genetic association remains controversial [11] and in some cases, increased resistance was observed in human carrying these variants [27]. Our sequence survey of 49 individuals confirms co-segregation of the high frequency D299G and T399I SNPs whose presence identifies our allele 1 in 5.1% of the cases (Table S3). Furthermore, we have identified an additional rare (1% among the alleles identified here) amino acid change (C281Y) in the TLR4 ectodomain. This variant, which was not detected in previous sequencing surveys of large cohorts [28], [29], was later identified in an exhaustive study of *TLR4* variations in meningococcal sepsis patients compared to ethnic matched controls [12]. This analysis revealed that neither the occurrence of common variants nor rare single SNPs were significantly associated with increased risk of disease. However, it was observed that, collectively, rare TLR4 variants were overrepresented in the patient population.

### *TLR5*

**Figure 4:**
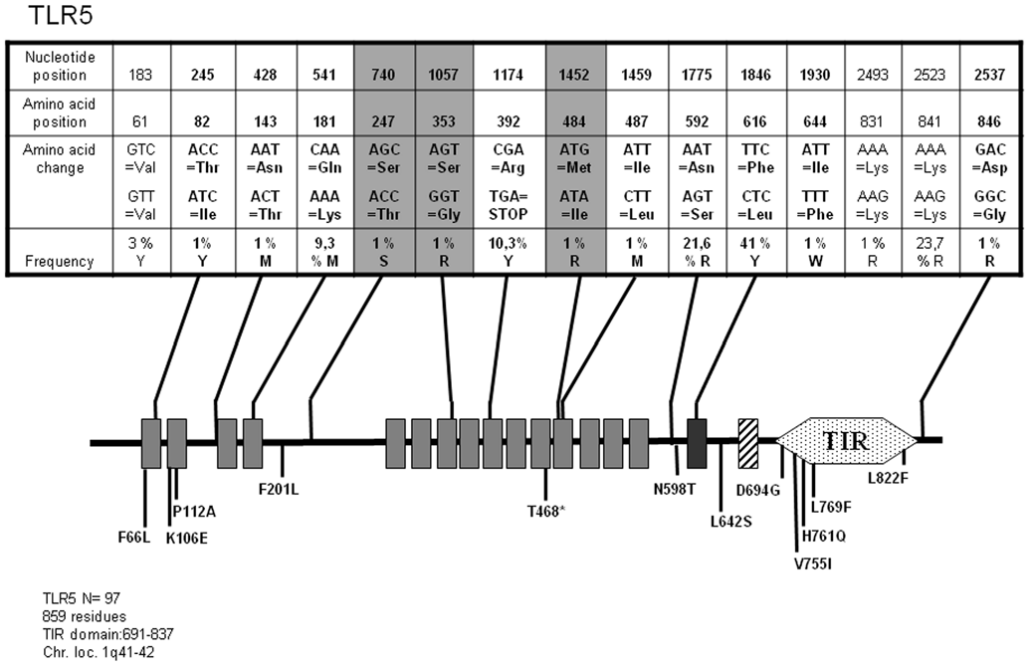
Schematic representation of TLR5 and position and frequency of Single Nucleotide Polymorphisms (SNPs). Legend is similar to Fig. 1.

TLR5 responds to bacterial flagellin [30]. Our analysis of the *TLR5* coding sequences in 97 unrelated individuals shows that we detected 9 non synonymous SNPs out of 21 recorded in the databases, including the frequent R392Stop, N592S and F616L (Fig. 4). In addition, we found 3 additional non-synonymous SNPs in the TLR5 ectodomain, two of which (S353G and M484I) are located within Leucine-rich repeats (LRR) and the fourth (S247T) outside of these domains. Altogether, the 15 *TLR5* SNPs that we detected in our cohort were grouped in 14 alleles whose translation generates 12 forms of the TLR5 protein (Table S4) among which those containing the new non-synonymous changes alone occur at low frequency (<1%). We also note that Q181K either segregates with both R392Stop (Allele 6, 4.2%) or with F616L (Allele 5 at a frequency of 0.5%).

### *TLR6*

**Figure 5:**
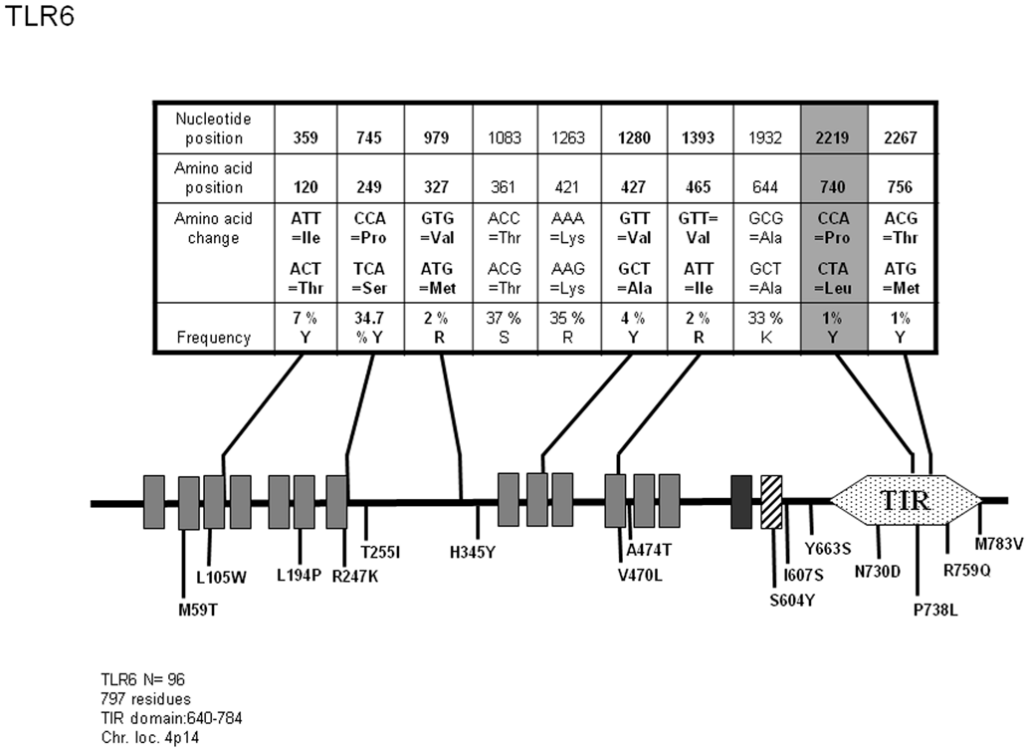
Schematic representation of TLR6 and position and frequency of Single Nucleotide Polymorphisms (SNPs). Legend is similar to Fig. 1.

As mentioned above, TLR6 interaction with TLR2 has been reported to be required for recognition of microbial determinants such as zymosan and Lipoteichoic acid (LTA) [31], [32]. In this study, we have detected 6 non-synonymous SNPs out of 21 which are already known and deposited in databanks. One new polymorphic position inducing an amino acid change was also observed (Fig. 5): P740L is located inside the TIR domain and thus, could modify signal transduction. The *TLR6* allelic repertoire, derived from sequences obtained from 96 unrelated donors is shown on Table S5. 10 alleles have been identified here at the nucleotide level, among which allele 6 expressing the P740L SNP is represented in 1.1% (denominated allele 4 when alleles are defined according to amino acids sequences) of TLR6 proteins in our population.

### *TLR7 and TLR8*

**Figure 6:**
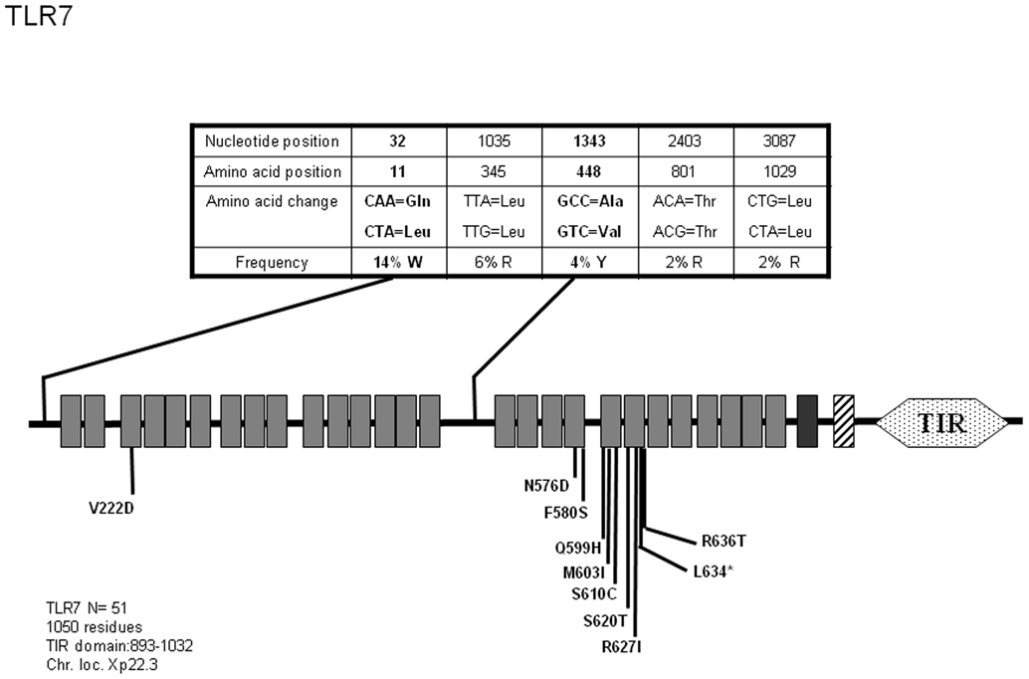
Schematic representation of TLR7 and position and frequency of Single Nucleotide Polymorphisms (SNPs). Legend is similar to Fig. 1.

**Figure 7:**
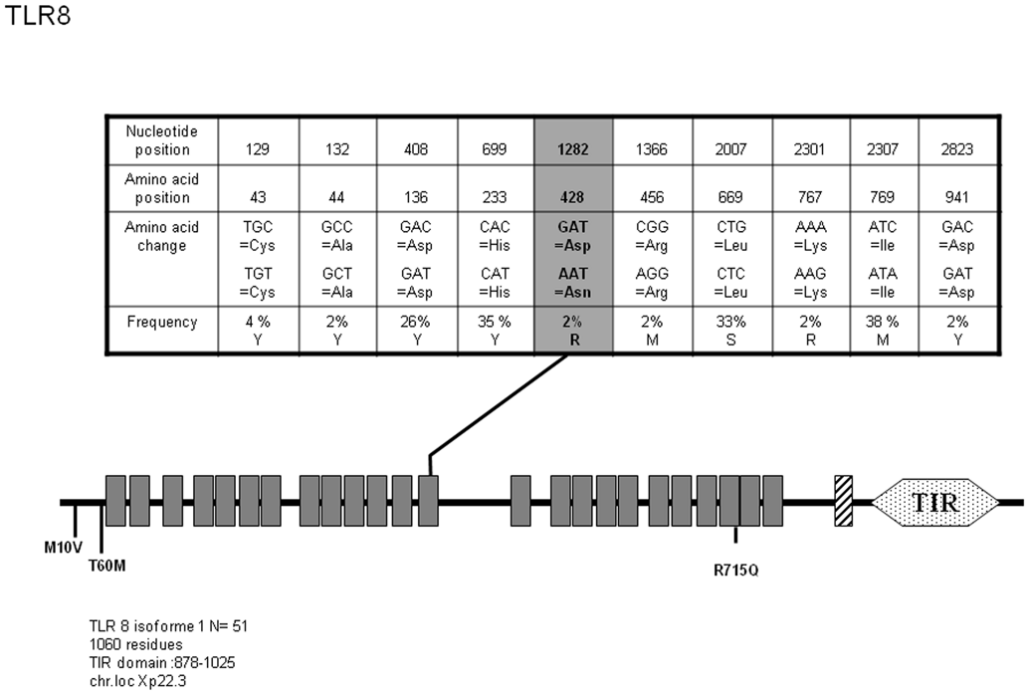
Schematic representation of TLR8 and position and frequency of Single Nucleotide Polymorphisms (SNPs). Legend is similar to Fig. 1.

These receptors are believed to bind viral and synthetic single-stranded RNAs as well as Imidazoquinoline-based small molecules and guanosine-based nucleosides [33], [34], [35]. This functional similarity is correlated to the high sequence homology between the two molecules and their specific subcellular localization (TLR7 and TLR8, but also TLR3 and TLR9 are expressed at the endosomal membrane). Our analysis of *TLR7* sequences in 51 individuals did not allow the identification of additional SNPs to those already recorded in databanks. As seen on Fig. 6, we detected 5 SNPs among which 2 are non-synonymous (Q11L and A448V). Allelic variants analysis (Table S6) indicates that Q11L is frequently represented in TLR7 mutant alleles (alleles 2 and 3, 17.5% combined) and in 2.5%, is associated to A448V mutation (allele 2). Comparatively, TLR8 shows very limited variation. Out of 15 reported SNPs, only 3 (which were not identified in this study) induce amino acid variation in the protein sequence. However, we have identified an additional non-synonymous SNP (D428N, see Fig. 7) which was observed in 2% of the DNA tested. This mutation induces the replacement of a negatively charged residue by a basic one and occurs in a Leucine-rich repeat (LRR), which may prompt further investigation (see discussion) given its location at the probable dimer interface (according to other TLRs crystallographic data). Allele classification (Table S7) shows that this modification segregates with additional SNPs which do not generate differences at the protein level (allele 7, 1% of the chromosomes). At the protein level, D428N identifies allele 2.

### *TLR9*

**Figure 8:**
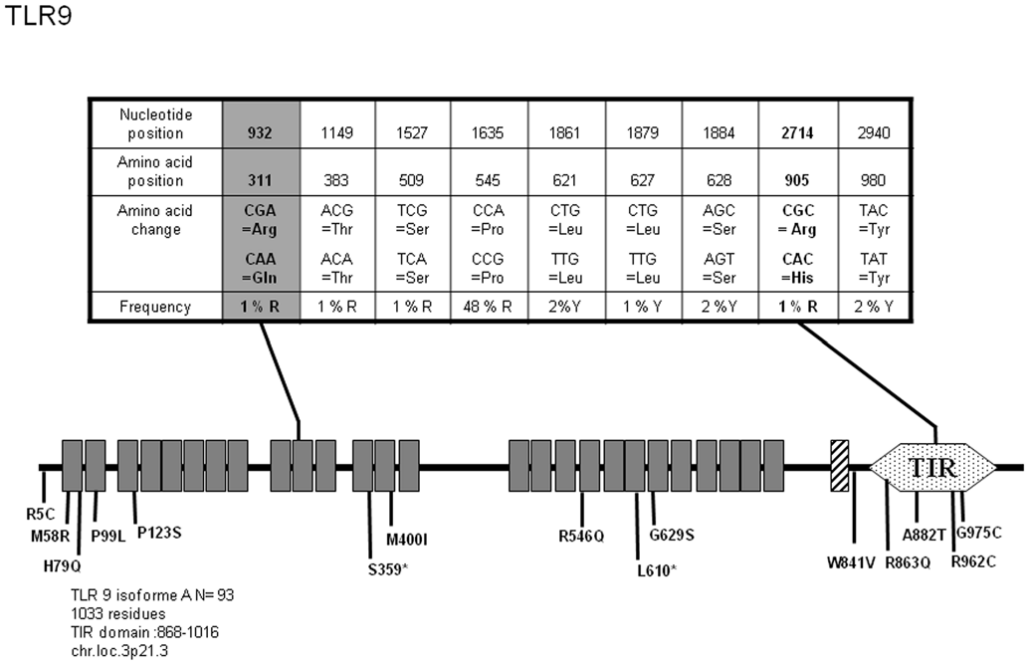
Schematic representation of TLR9 and position and frequency of Single Nucleotide Polymorphisms (SNPs). Legend is similar to Fig. 1.

The discovery of unmethylated (prokaryotic) DNA as the ligand for TLR9 [36] has fuelled several hypotheses regarding the likely involvement of this receptor in innate immune defence. Few genetic association studies have evaluated the linkage between *TLR9* SNPs and infectious diseases. Recently, rapid progression phenotype following HIV infection [37] and malaria manifestation during pregnancy [38] were both traced to TLR9 polymorphic positions. It was also suggested that the function of TLR9 in B cell response to autoantigens and in dendritic cell response to chromatin immune complexes may have an effect on susceptibility to SLE. Yet, a recent evaluation of this possibility provided negative results [39]. So far, the most studied TLR9 SNP (C-1237T), which has been linked to asthma [40], is located within the promoter region of the *TLR9* gene. It is therefore surprising to note that among the 16 non-synonymous SNPs in the *TLR9* coding sequence and recorded in public databases (see Fig. 8), none has been assessed so far in relation to any pathology. Our *TLR9* sequencing survey in 93 donors has led to the discovery of an additional non-synonymous SNP inducing a modification in a LRR located in the central part of the ectodomain (R311Q) which, by its sole presence, define a specific allele (allele 3 in Table S8). According to the location of the mutation and by analogy with published TLR structures [21], [41], [42], this TLR9 allele might exhibit defects in dimer formation and/or ligand recognition. Translation of the different nucleotide sequences predicts 3 TLR9 proteins, among which each mutated form (allele 2, R863Q and allele 3, R311Q) represents 0.5%.

### *TLR10*

**Figure 9:**
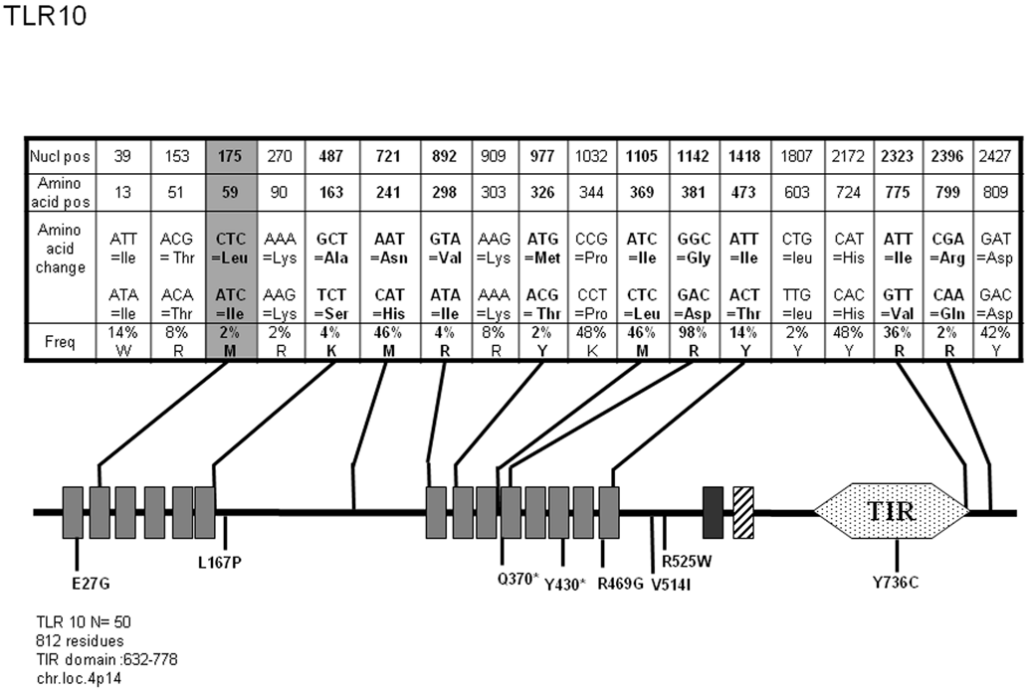
Schematic representation of TLR10 and position and frequency of Single Nucleotide Polymorphisms (SNPs). Legend is similar to Fig. 1.

TLR10 is the last Toll-like Receptor discovered in man [43] and since then, it has remained an orphan member of this family. TLR10 shares with TLR1 and TLR6 a common locus on chromosome 4p14 and the three TLRs are structurally similar to one another. Resequencing of TLR10 in 47 subjects has identified most SNPs and suggested possible association of this gene with asthma [44]. In this study, TLR10 was sequenced for 50 donors. As illustrated Fig. 9, we identified most polymorphic positions in the coding sequence (18 out of a total of 29 which are reported) among which 9 SNPs induce amino acid change. We have also detected one new non-synonymous SNP (L59I) in the second LRR of the ectodomain. Table S9 lists the 15 TLR10 alleles, among which allele 9 expressing the single L59I mutation represents 1.32% of the repertoire.

## Discussion

Susceptibility to infections is heritable, as demonstrated in a seminal study which examined and compared the cause of death among adoptees to that of their biological or adoptive parents [45]. Although many immunodeficiencies have been mapped to a single locus (monogenic diseases, such as X-linked or autosomal severe combined immunodeficiencies), until recently, a role for TLR in susceptibility to infections had not firmly been assessed. Two genetic studies have demonstrated the involvement of downstream components used by the TLR pathway: IRAK4 mutations have been linked to increased infections by pyogenic bacteria [46] and UNC-96B defects in two children was associated to HSV-1 encephalitis [47]. The same group has now identified a *TLR3* mutation in humans and reports for the first time HSV-1 encephalitis in children carrying the mutated allele [48]. This contrasts with the wealth of information which was produced with animal models in which experimental infections in mice have been instrumental in defining the TLR specificities. Despite high homologies between mouse and human genes, it is now clear that human genetic diversity (as opposed to genetic homogeneity in mouse models) and environmental conditions which may or may not bring in close proximity a pathogen and its host, render human genetic studies, in addition to the development of animal models, a required step to understand immune responses to natural infections. To contribute to this goal, we have resequenced *TLR1–10* (but not TLR3 which was excluded from the analysis for technical reasons, see Materials and Methods) in up to one hundred unrelated individuals of Caucasian origin, and “scanned” all the synonymous and non-synonymous SNPs in the coding sequence. Because we focused our study to the coding sequences of TLRs, we concentrated our present analysis on non-synonymous SNPs, which can introduce modifications potentially affecting the function of the corresponding proteins. Nevertheless, we also recorded synonymous SNPs and compared them to those which are already described in public databases. From this effort, several points deserve to be highlighted. (i) First, the general picture emerging from the comparison of TLR1–10 allelic repertoire is that these molecules are not equally polymorphic. Clearly, TLR10, 5, 1 and 6 show the highest level of variation in our Caucasian sample with respectively 15, 12, 10 and 8 possibilities for the encoded proteins. Only 3 non-synonymous SNPs we detected for TLR4, whereas 29 are deposited in databanks. The limited number of alleles identified in our study likely reflects the lowest amount of samples which were analysed, compared to more extensive investigations [28], [29]. On the contrary, besides this sub-group of highly variable TLRs, TLR 7, 8 and 9 appear less flexible. We detected only two known non-synonymous SNPs in TLR7 (out of 12 which are recorded), one in TLR8 (in addition to 3 known non-synonymous SNPs) and two in the TLR9 coding sequence (out of 16 already described). We speculate that selection pressure prevents mutations in these genes because the proteins encoded by these TLRs recognize nucleic-acid-based structures which are highly conserved and subjected to almost no possible variation in their size, charge or other physicochemical features. Conversely, evolutionary constrains have likely shaped TLR1, 4, 5 and 6 to render them able to detect a whole panel of pathogens, despite expression of highly variable molecules such as LPS and lipoproteins among different microorganisms. In this regard, TLR2 limited variation remains enigmatic, but the multiple alleles of the still orphan TLR10 suggests that this molecule, possibly associated with TLR1 or TLR2, likely participates in bacterial determinants sensing. (ii) Second, several new non-synonymous polymorphic positions were identified in this study (I57M, L443I, V542A and T565S in TLR1, S247T, S353G and M484I for TLR5; D428N in TLR8; R311Q in TLR9 and L59I in TLR10). All these positions are located in the ectodomain of the TLRs which contains the LRR and could therefore potentially affect ligand binding and/or dimer formation. Future studies, using TLR expression vectors transfected in culture cells, should clarify the effect of these mutations on the functionality of these receptors. In this respect, it is interesting to note that such studies designed to quantify the impact of specific SNPs in TLRs have been performed only in few instances [24]. (iii) Third, our effort, combined with those of others, is important to obtain reliable sequence and SNP information, which ultimately, are validated by comparing data from multiple sources. For instance, we observed that the so-called S248N variant of TLR1 is present in 3 alleles (alleles 5, 6 and 7; Table S1) with a combined frequency of 70.24%. In agreement with others [49], our data indicate that Asn (N) residue at position 248 rather represents the normal form of TLR1 and its replacement by a Ser (S) identifies a variant form. (iv) Lastly, we wish to emphasize that our effort provided not only frequencies for individual SNPs in the *TLR* coding sequences, but also afforded an overall picture of the allelic repertoire for this important class of proteins. Resequencing is a useful approach to assemble SNPs at the level of a single gene and, as pointed by others [50], [51], is more powerful than single SNP analysis to analyse complex traits. Because recombination between SNPs may affect gene variability, our description of all the alleles in a single cohort provides valuable information regarding the heterogeneity of *TLR* genes in an ethnically-defined population. It should also contribute to clarify the effect of single SNPs in TLRs which were not convincingly replicated in several independent investigations (see [52] for a recent update on TLR polymorphisms linked to human disease).

In conclusion, because SNPs (and in some cases, their combination in rare alleles) may play a decisive role in common diseases, knowledge of allelic repertoire of genes involved in immune responses is an essential step toward the understanding of the genetic grounds of infectious or auto-immune pathologies. The impressive diversity of MHC-related genes provided essential clues to this problem, but the re-evaluated importance of innate immunity in the defence against pathogens has recently stimulated investigations designed to thoroughly describe pattern recognition receptors (PRR) such as TLRs at the genetic level in humans. Altogether, our data, which improve the picture of TLR diversity in humans, contribute to this endeavour. As such, they provide preliminary foundation for a future effective genetic analysis enabling their inclusion in “personalized” or “individualized” predictive medicine as related to the immune function.

## Materials and Methods

### Origin of genomic DNA

DNA samples arise from 100 anonymous Caucasian blood donors from the Strasbourg, France area, in accordance with the actual French legislation at the time of collection.

### PCR amplification, cloning and sequencing

In most cases, PCR reactions were performed using the Expand Long Template PCR system (Roche Diagnostics) using 400 ng of genomic DNA according to the manufacturer's specifications except for the annealing temperature and in some cases for the number of cycles (Table S10). For TLR4, shorter exons were individually amplified using standard Taq DNA Polymerase (Promega, WI, USA) using 200 ng of genomic DNA. All primers were designed by aligning cDNA sequences with genomic (chromosomal) sequences available in public databases (NCBI and Ensembl). The PCR products were purified using the Nucleospin® extract purification kit (Macherey-Nagel) and sequenced using ABI PRISM BigDye terminator v3.0 Cycle Sequencing Ready Reaction Kit® with AmpliTaq DNA Polymerase, FS (Applied Biosystems Warrington, UK). Sequencing primers were designed in order to cover an average of 450 bp. Reactions were performed using 2 µl of Sequencing mix, 6.4 pmol of primers, 150–200 ng of PCR products in a total volume of 10 µl. The reactions were run on an ABI 3100 DNA Sequencing System and analyzed using Applied Biosystem's SeqScape® software using published sequences as reference. Using several combinations of primer pairs, we could not successfully amplify the totality of TLR3, neither using long-range technology, we equally failed to amplify each and all exons individually. Not being able to gain information on the totality of the sequence we thus excluded TLR3 from this study. Every PCR reaction was performed in duplicate. One was used for direct sequencing and the other, if necessary (when a heterozygous position was detected upon direct sequencing of the amplicon) served to clone the fragment. Cloning was performed using the TA cloning kit® (Invitrogen) according to the manufacturer's protocol. For each construction, 12 clones were purified and sequenced, which enabled in most case the identification of the 2 alleles from the donor. Nucleotide sequence data of the allelic variants reported in this article are available in the NCBI database under the accession numbers given in Table S11.

### SNP and allelic frequencies

SNP frequency is calculated as the occurrence of a specific heterozygote position in relation to the total number of samples (N) (or donors, necessarily diploid) for which a given TLR was successfully sequenced (see text for the amount of successful PCR for each TLR). Allelic frequency is reported to the number of chromosomes (2N) which were sequenced.

## Supporting Information

Table S1 Frequency of the TLR1 alleles at the nucleotidic (top) and proteic (bottom) level. Bold letters indicate Non Synonymous Single Nucleotide Polymorphisms (NS-SNPs).
(0.01 MB XLSX)

Table S2 Frequency of the TLR2 alleles at the nucleotidic (top) and proteic (bottom) level. Bold letters indicate Non Synonymous Single Nucleotide Polymorphisms (NS-SNPs).
(0.01 MB XLSX)

Table S3 Frequency of the TLR4 alleles at the nucleotidic (top) and proteic (bottom) level. Bold letters indicate Non Synonymous Single Nucleotide Polymorphisms (NS-SNPs).
(0.01 MB XLSX)

Table S4 Frequency of the TLR5 alleles at the nucleotidic (top) and proteic (bottom) level. Bold letters indicate Non Synonymous Single Nucleotide Polymorphisms (NS-SNPs).
(0.01 MB XLSX)

Table S5 Frequency of the TLR6 alleles at the nucleotidic (top) and proteic (bottom) level. Bold letters indicate Non Synonymous Single Nucleotide Polymorphisms (NS-SNPs).
(0.01 MB XLSX)

Table S6 Frequency of the TLR7 alleles at the nucleotidic (top) and proteic (bottom) level. Bold letters indicate Non Synonymous Single Nucleotide Polymorphisms (NS-SNPs).
(0.01 MB XLSX)

Table S7 Frequency of the TLR8 alleles at the nucleotidic (top) and proteic (bottom) level. Bold letters indicate Non Synonymous Single Nucleotide Polymorphisms (NS-SNPs).
(0.01 MB XLSX)

Table S8 Frequency of the TLR9 alleles at the nucleotidic (top) and proteic (bottom) level. Bold letters indicate Non Synonymous Single Nucleotide Polymorphisms (NS-SNPs).
(0.01 MB XLSX)

Table S9 Frequency of the TLR10 alleles at the nucleotidic (top) and proteic (bottom) level. Bold letters indicate Non Synonymous Single Nucleotide Polymorphisms (NS-SNPs).
(0.01 MB XLSX)

Table S10 Oligonucleotides and PCR conditions used for analysis of TLR genes.
(0.01 MB XLSX)

Table S11 Genbank accession numbers for all TLR alleles described in this study.
(0.01 MB XLSX)
